# Active Inflammatory and Chronic Structural Damages of Sacroiliac Joint in Patients With Radiographic Axial Spondyloarthritis and Non-Radiographic Axial Spondyloarthritis

**DOI:** 10.3389/fimmu.2021.700260

**Published:** 2021-07-27

**Authors:** Liudan Tu, Churong Lin, Ya Xie, Xiaohong Wang, Qiujing Wei, Yanli Zhang, Jieruo Gu

**Affiliations:** ^1^Department of Rheumatology, Third Affiliated Hospital of Sun Yat-Sen University, Guangzhou, China; ^2^Department of Radiology, Third Affiliated Hospital of Sun Yat-Sen University, Guangzhou, China

**Keywords:** ankylosing spondylitis, axial spondyloarthritis, inflammation, structural damage, MRI

## Abstract

**Objective:**

Evaluate the MRI evidence of active inflammatory and chronic structural damages in radiographic axial spondyloarthritis (r-axSpA) and non-radiographic axial spondyloarthritis (nr-axSpA).

**Methods:**

A retrospective review of 253 patients who underwent sacroiliac joint (SIJ) MRI between June 2014 and December 2019 was performed. MRI images including short tau inversion recovery scan and T1-weighted spin echo scans were assessed using the Spondyloarthritis Research Consortium of Canada (SPARCC) score and SPARCC MRI SIJ structural score by two independent readers.

**Results:**

Higher mean score of inflammatory (SPARCC) was seen in r-axSpA patients when compared with nr-axSpA patients (8.08 *vs* 4.37, *P*<0.05). Frequencies of MRI structural lesions in r-axSpA patients and nr-axSpA patients were as follows: erosion (65.84 *vs* 88.23%, *P*=0.002), backfill (33.17 *vs* 13.73%, *P*<0.001), fat metaplasia (79.21 *vs* 60.78%, *P*=0.01), and ankylosis (37.13 *vs* 1.96%, *P*<0.001). Patients with r-axSpA had a higher mean score for fat metaplasia (8.93 *vs* 4.06, *P*=0.0003) and ankylosis (4.49 *vs* 0.04, *P*<0.001).

**Conclusion:**

More active inflammatory and chronic structural damages except for erosion were seen in r-axSpA patients than nr-axSpA patients, while higher percentage of nr-axSpA patients presented with erosion in MRI.

## Introduction

Spondyloarthritis (SpA) is a group of inflammatory diseases mainly affecting the sacroiliac (SI) joint and spine. Axial spondyloarthritis (axSpA) includes non-radiographic axial spondyloarthritis (nr-axSpA) and radiographic axial spondyloarthritis (r-axSpA); the latter one is also termed ankylosing spondylitis (AS). Nr-axSpA and r-axSpA share some similarities such as disease activity (defined by Bath Ankylosing Spondylitis Disease Activity Index—BASDAI), physical function, prevalence of HLA-B27 positivity, and comorbidity burden (1–3), while there are several differences including disease course, gender predilection, and levels of inflammatory markers (4).

MRI is an objective measurement detecting acute inflammatory and chronic structural changes recommended by the Assessment of SpondyloArthritis International Society (5). Bone marrow edema (BME), capsulitis, and subligamentous enthesitis are defined as active inflammatory changes in SpA according to the update of the ASAS MRI working group (6). Chronic structural changes including subchondral sclerosis, erosion, backfill, fat metaplasia, and ankylosis are present better and earlier in CT (7) or MRI (8) than radiographs.

Inflammatory and chronic structural changes are objective signs of axSpA. Previous studies indicated that sacroiliitis on radiographs is usually bilateral and symmetric in AS (9). However, the differences of structural damages between r-axSpA and nr-axSpA patients on MRI are limited. The aim of this study was to investigate whether there are differences between Chinese r-axSpA and nr-axSpA patients in inflammatory and chronic structural lesions on MRI, and clinical factors related to MRI changes.

## Methods

### Patients

A retrospective study was performed, and patients with MRI and radiographic imaging of the sacroiliac joint and diagnosed with axSpA between June 2014 and December 2019 were included. The local ethics committee waived approval because of the retrospective nature of this study. The patients provided their written informed consent to anonymize the use of relative data in further study according to daily clinical practices in this department. Demographic characteristics and inflammatory indicators such as C-reactive protein (CRP) and Erythrocyte Sedimentation Rate (ESR) are collected.

### MRI Protocol and Assessment

**Figure 1:**
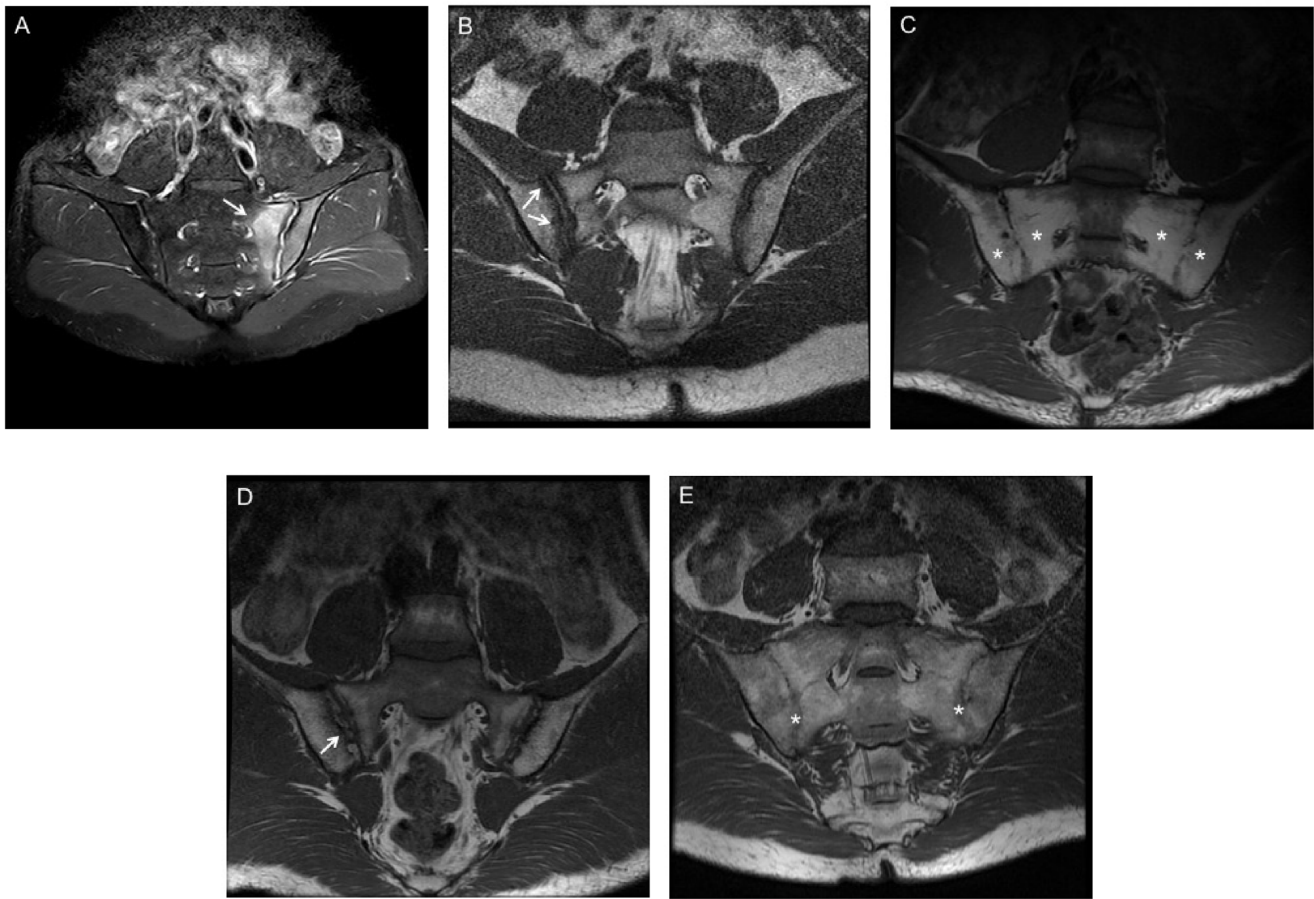
MRI findings of inflammatory and structural damage of SpA. **(A)** Bone marrow edema (BME) at the left side of sacroiliac joints (white arrows) in Coronal oblique STIR sequence. **(B)** Erosion of the right iliac bone on T1 weighted spin echo MRI (white arrows). **(C)** Fat metaplasia at the bilateral sacroiliac joints on T1 weighted spin echo MRI (white stars). **(D)** Backfill of the right iliac bone on T1 weighted spin echo MRI (white arrows). **(E)** Ankylosis of the bilateral sacroiliac joints on T1 weighted spin echo MRI (white stars).

All MR images were acquired on a 1.5 Tesla or 3 Tesla MR scanner in a coronal plane titled parallel to the long axis of the SI joint with 3–4 mm slice thickness and 12–15 slices acquired. Short-tau inversion recovery (STIR) sequences were used for the assessment of inflammatory lesion. The T1-weighted MRI sequences were used for the assessment of chronic/structural changes. The Spondyloarthritis Research Consortium of Canada SPARCC MRI scoring system was used to assess inflammation and structural damages in SI joint. SPARCC SIJ scores were based on the measurement of six consecutive slices, with the largest score of 12 for edema, intensity, and depth per slice, and a total score of 0–72 was calculated. SPARCC SI structural lesion score (SSS) (10) was used to assess the structural lesions, and four kinds of lesions were assessed based on five consecutive slices through the SIJ. Fat metaplasia is defined as an increased signal in bone marrow on T1WSE, and the lesion has to demonstrate homogeneous signal with more than 1 cm in depth from the joint surface. Erosion is defined as the full-thickness loss of the dark appearance of either iliac or sacral cortical bone at its anticipated location and loss of the normal bright appearance of adjacent bone marrow. Backfill is defined as complete loss of iliac or sacral cortical bone at its anticipated location and increased signal that is clearly demarcated from adjacent normal marrow by irregular dark signal reflecting sclerosis at the border of the eroded bone. Ankylosis is defined as bone marrow signal on T1WSE sequences extending between the sacral and iliac bone marrow with a full-thickness loss of the dark appearance of the iliac and sacral cortical bone. The presence/absence of lesions is scored using an online data entry system in SIJ quadrants (fat, erosion) or halves (backfill, ankylosis) with a scoring range of 0–40 for quadrants lesions and 0–20 for halves lesions. Examples of these inflammatory and structural damages are shown in **Figure 1**. Images were assessed by two radiologists independently, who were blinded to all patient data. The mean score of two radiologists was used for analysis. For SPARCC SIJ score, two readers had to discuss and reach consensus if a difference of >3 points existed (11). A SPARCC SSS >0 was required from both readers for identification of a structural lesion of erosion, backfill, fat metaplasia, or ankylosis (12). The before image evaluation, a training session including 20 test images, was carried out by an expert rheumatologist. A score ≥2 for SI joint bone marrow edema (BME) was considered positive MRI evidence of inflammation, and this cutoff has been validated in the literature (3, 13).

### Statistical Analysis

Statistical analyses were performed using Stata Version 15.0. Summary statistics such as frequency, percentage, mean, and standard deviation were used to analyze demographic characteristics and inflammatory and structural changes of axSpA patients. T-test was used to evaluate the difference of demographic characteristics and MRI scores between AS and nr-axSpA patients. Spearman correlations were used to determine the correlation between SPARCC scores and clinical factors. Then we conducted multivariate stepwise regression analyses to determine the best significant predictors for inflammatory and structural lesions. All tests were two-sided, and a significance level of *P* < 0.05 was assumed. Interobserver reliability for imaging scores was assessed using the intraclass correlation coefficient (ICC). The ICC value of <0.4 was designed as fair, ≥0.4 but <0.6 as moderate, ≥0.6 but <0.8 as good, ≥0.8 but <0.9 as very good, and ≥0.9 as excellent.

## Results

### Demographic

A total of 253 patients with a clinical diagnosis of axSpA were included for analysis during June 2014 and December 2019 in the third affiliated hospital of SUN YAT-SEN University. Fifty-one patients were classified as nr-axSpA, while others fulfilled the diagnosis of r-axSpA. The average age of patients was 31.74 years with a mean disease duration of 7.91 years. A total of 87.35% (n=221) of patients were HLA-B27 positive, and 79.45% of them were male. The mean BASDAI and BASFI scores were 4.31 ± 2.25 and 2.19 ± 2.27 respectively, and 33.2% (n=84) of patients had used tumor necrosis factor inhibitor (TNFi) in the recent 3 months. Longer disease duration, higher positive rate of HLA-B27, and worse function, as measured by BASFI, were seen in r-axSpA group. The disease activity markers of BASDAI, ASDAS, and SPARCC MRI SIJ inflammation were significantly higher in r-axSpA group. No significant differences in other demographic features were noted between the groups (**Table 1**).

**Table 1:** Demographic features of included patients.

Characteristics	All patients (n=254)	AS (n=202)	nr-axSpA (n=51)	*P*-value
Age (years)	31.74±9.14	31.7±9.25	31.88±8.77	0.9
Male patients, n (%)	201 (79.45)	164 (81.19)	37 (72.55)	0.18
Disease duration (years)	7.91±6.56	8.68±6.50	4.94±5.95	0.0002
HLA-B27, n (%)	221 (87.35%)	182 (90.1)	39 (76.47)	0.02
Family history, n (%)	60 (23.72)	51 (25.25)	9 (17.65)	0.35
CRP (mg/dl)	18.66±23.99	20.05±25.18	13.19±17.75	0.06
ESR (mm/H)	26.07±24.13	26.54±23.22	24.18±27.58	0.53
BASDAI (0-10)	4.31±2.25	4.53±2.26	3.43±2.02	0.002
BASFI (0-10)	2.19±2.27	2.38±2.29	1.44±0.05	0.009
ASDAS-CRP	2.98±1.17	3.14±1.13	2.37±1.13	<0.000
Use of TNFi, n (%)	84 (33.2%)	63 (31.2%)	21 (41.2%)	0.19

Data were presented as mean±SD unless specifically indicated. HLA-B27, Human leukocyte antigen-B27; CRP, C-Reactive Protein; ESR, Erythrocyte Sedimentation Rate; BASDAI, Bath Ankylosing Spondylitis Disease Activity Index; BASFI, Bath Ankylosing Spondylitis Functional Index; BASMI, Bath Ankylosing Spondylitis Metrology Index; ASDAS, Ankylosing Spondylitis Disease Activity Score; TNFi, Tumor necrosis factor inhibitor.

### Inflammatory and Structural Changes on MRI

Higher SPARCC score was seen in r-axSpA patients than in nr-axSpA patients. Of the 253 patients evaluated, 117 (46.25%) had no BME seen on MRI. There was no significant difference in percentage of patients with BME between r-axSpA and nr-axSpA groups. Clinical characteristics such as age, sex, BASDAI, and CRP did not differ between patients with and without BME. There was significant difference in the mean score of erosion, backfill, and ankylosis between patients with and without SIJ BME (**Table 2**). Relative frequencies of MRI structural lesions in patients with *versus* without SIJ BME were as follows: erosion (78/136, 57.35% *vs* 100/117, 85.47%; *P*<0.001), backfill (23/136, 16.91% *vs* 51/117, 43.59%; *P*<0.001), fat metaplasia (93/136, 68.38% *vs* 98/117, 83.76%; *P*=0.005), and ankylosis (50/136, 36.76% *vs* 26/117, 22.22%; *P*=0.01).

**Table 2:** MRI inflammatory and structural damages.

Characteristics	All patients (n=253)	AS (n=202)	nr-axSpA (n=51)	BME≥2 (N=136)	BME<2 (N=117)
SPARCC (0-72)	7.33 ± 12.02	8.08 ± 12.27^*^	4.37 ± 10.55	–	–
Fat metaplasia (0-40)	7.95 ± 8.74	8.93 ± 9.15^*^	4.06 ± 5.33	7.56 ± 9.34	8.41 ± 7.98
Erosion (0-40)	5.01 ± 6.41	5.13 ± 6.79	4.51 ± 4.60	3.36 ± 5.39^η^	6.93 ± 6.97
Backfill (0-20)	1.51 ± 3.33	1.71 ± 3.35^*^	0.75 ± 3.19	0.68 ± 2.19^η^	2.48 ± 4.11
Ankylosis (0-20)	3.59 ± 6.72	4.49 ± 7.25^*^	0.04 ± 0.28	5.37 ± 7.98^η^	1.65 ± 4.10

SPARCC, the Spondyloarthritis Research Consortium of Canada; AS, ankylosing spondylitis; nr-axSpA, non-radiographic axial spondyloarthritis; BME, bone marrow edema. *Significant difference was found between AS and nr-axSpA patients.^η^Significant difference was found between patients with and without BME.

The majority of patients had a score of >0 for structural damages for at least one of fat metaplasia, erosion, backfill, and ankylosis in the SI joint. A total of 242 (95.65%) patients had ≥1 structural lesion on MRI comprising erosion 71.54%, backfill 29.24%, fat metaplasia 75.49%, and ankylosis 30.04%. In SSS structural scores, significant difference was found between r-axSpA and nr-axSpA groups in fat metaplasia, backfill, and ankylosis (**Table 2**). Frequencies of MRI structural lesions in r-axSpA patients and nr-axSpA patients were as follows: erosion (65.84 *vs* 88.23%, *P*=0.002), backfill (33.17 *vs* 13.73%, *P*<0.001), fat metaplasia (79.21 *vs* 60.78%, *P*=0.01), and ankylosis (37.13 *vs* 1.96%, *P*<0.001).

### Correlations Between MRI Inflammatory and Structural Lesions

Significant correlation was found between SPARCC score and age (−0.21, *P <*0.01), sex (−0.15, *P* =0.02). In multivariable stepwise regression analysis, age (−0.28, *P* =0.001) and HLA-B27 (4.53, *P* =0.046) were found significantly associated with SPARCC score.

**Table 3** shows factors associated with SSS scores. Fat metaplasia was associated with age, sex, and BASDAI, and only BASDAI (β: −0.56, *P* =0.02) remained significant in multivariable analysis. For erosion, significant association was found with BASDAI (β: −0.88, *P <*0.01) and SPARCC (β: 0.18, *P <*0.01) in multivariable analysis. Backfill was associated with SPARCC (β: 0.05, *P <*0.01) in multivariable analysis. For ankylosis, age, sex, HLA-B27, disease duration, CRP, and SPARCC were found significantly associated.

**Table 3:** Factors associated with structural damages on MRI.

	Fat metaplasia		Erosion		Backfill		Ankylosis	
	Univariable	Multivariable	Univariable	Multivariable	Univariable	Multivariable	Univariable	Multivariable
	Spearmen’s rho, P value	β (95% CI)	Spearmen’s rho, P value	β (95% CI)	Spearmen’s rho, P value	β (95% CI)	Spearmen’s rho, P value	β (95% CI)
Age	**0.12, P=0.046**	NA	-0.08, NS	NA	-0.07, NS	NA	-0.02, NS	**-0.17 (-0.27, -0.07)**
Sex	-0.02, NS	NA	0.10, NS	NA	-0.007, NS	NA	**-0.15, P=0.02**	**-2.14 (-3.83, -0.44)**
HLA-B27	0.11, NS	3.09 (-0.13, 6.30)	-0.02, NS	NA	0.09, NS	NA	**0.15, P=0.02**	**2.50 (0.16, 4.83)**
Disease duration	**0.14, P=0.02**	NA	**-0.12, P=0.48**	NA	-0.02, NS	NA	**0.26, P<0.001**	**0.27 (0.13, 0.41)**
BASDAI	**-0.16, P<0.01**	**-0.88 (-1.21, -0.54)**	**-0.39, P<0.01**	**-0.88 (-1.21, -0.54)**	-0.10, NS	NA	0.12, NS	NA
CRP	0.04, NS	NA	**-0.13, P=0.04**	NA	-0.02, NS	NA	**0.28, P<0.001**	**0.05 (0.02, 0.08)**
ESR	-0.05, NS	NA	-0.06, NS	NA	0.006, NS	NA	0.12, NS	NA
ASDAS	-0.07, NS	NA	**-0.31, P<0.01**	NA	-0.07, NS	NA	**0.22, P<0.001**	NA
SPARCC	0.09, NS	NA	**0.36, P<0.01**	**0.18 (0.12, 0.24)**	**0.31, P<0.001**	**0.05 (0.01, 0.09)**	**-0.22, P<0.01**	**-0.17 (-0.23, -0.10)**

HLA-B27, Human leukocyte antigen-B27; BASDAI, Bath Ankylosing Spondylitis Disease Activity Index; BASFI, Bath Ankylosing Spondylitis Functional Index; CRP, C-Reactive Protein; ESR, Erythrocyte Sedimentation Rate; ASDAS, Ankylosing Spondylitis Disease Activity Score; SPARCC, the Spondyloarthritis Research Consortium of Canada; NS, not significant; NA, not available.

Bold values means significant difference.

### Interobserver Reliability

ICC was computed to test interobserver reliability. Excellent correlation was achieved for rating of SPARCC score and ankylosis (ICC scores of 0.98 [95% CI 0.93–0.99] and 0.95 [95% CI 0.91–0.98], respectively). Good correlation was achieved for identification of erosion, fat metaplasia, and backfill with ICC scores of 0.85 (95% CI 0.71–0.93) for fat metaplasia, 0.87 (95% CI 0.75–0.94) for erosion, and 0.74 (95% CI 0.52–0.87) for backfill.

## Discussion

The results of this study demonstrate that structural lesions on MRI may occur in early stage of SpA (nr-axSpA) and in the absence of SIJ BME on MRI. R-axSpA patients have more SIJ inflammation on MRI, and fat metaplasia is seen more in r-axSpA than nr-axSpA patients. Our data also support that structural damages in SIJ are associated with active inflammation.

Conventional radiograph is still preferred for the analysis of SIJ structural damage and for the diagnosis of axSpA. It has been verified that T1-weighted MRI sequences were superior to conventional radiographs in the detection of erosion of SIJ in axSpA patients (14). Besides, additional lesions such as fat metaplasia, backfill, and ankylosis can be observed clearly on MRI. The distribution pattern of inflammation and structural lesions may differ between AS and nr-axSpA patients; unilateral fat metaplasia and erosion were more seen in nr-axSpA patients (15) with a small simple size. This study compared the inflammation and structural lesions in r-axSpA and nr-axSpA patients using SPARCC scoring system and analyzed factor related to MRI changes.

Fat metaplasia, one of the chronic lesions and usually observed in the bone marrow of SI joint and spine, is assumed to be an intermediate stage between active inflammation and formation of new bone (16). Prospective studies indicated that fat metaplasia and backfill are associated with the development of new ankylosis (8, 14, 16). However, the proportion of fat marrow increases over time, and a previous study reported that a very high prevalence of fat metaplasia (50.6% in the age groups less than 45years, 94.4% in patients ≥75 years) was found in the SIJ of asymptomatic patients, while erosions were extremely uncommon (17). Fat metaplasia with certain features such as a distinct border, proximity to subchondral bone, and homogeneity of T1-weighted signal, combined with other abnormalities (bone marrow edema and erosion), is assumed to be highly specific for SpA but does no help for diagnosis (18).

The relationship between inflammation, fat metaplasia, and ankylosis has been studied before. It is reported that baseline SPARCC SIJ inflammation and SIJ backfill scores independently predicted progression in SPARCC SIJ ankylosis (19). TNFi can retard not only inflammation but also structural progression rate on MRI in both short-term (11, 20) and long-term follow-up (19). It is reported that etanercept has more effect than usual care on SIJ erosion development; attaining sustained ASDAS inactive disease is relevant to the amelioration of erosion (21). Reduction of inflammation, erosion, and increase of fat metaplasia were seen in SpA patients treated with adalimumab compared with placebo group (20). Other clinical factors such as disease activity, demographics, and HLA-27 were not associated with development of MRI structural features according to previous study (22).

There are several limitations of this study. First is the retrospective nature, which is subject to inherent observation bias. Second is the interpretation of the MRI T1 sequence. The structural damages such as erosion and backfill are heterogeneous lesions, which are difficult to discern and need caution when interpreting the results. Besides, these damages could be presented in other diseases such as scoliosis and degenerative disease. Further validation of structural damages using both computed tomography and MRI is needed for better understanding.

## Conclusions

This study indicated that higher inflammatory and structural damages except for erosion were seen in r-axSpA patients when compared with nr-axSpA patients. In patients without MRI SIJ BME, structural lesions are also present. Prospective study is necessary to understand the relationship between structural damages and treatments.

## Data Availability Statement

The original contributions presented in the study are included in the article/supplementary material. Further inquiries can be directed to the corresponding author.

## Ethics Statement

The studies involving human participants were reviewed and approved by third affiliated hospital of SUN YAT-SEN University. The patients/participants provided their written informed consent to participate in this study.

## Author Contributions

JG had full access to all of the data in the study and takes responsibility for the integrity of the data and the accuracy of the data analysis. Study concept and design: JG and LT. Acquisition, analysis, or interpretation of data: all authors. Drafting the manuscript: LT and JG. Critical revision of the manuscript for important intellectual content: all authors. All authors contributed to the article and approved the submitted version.

## Funding

This study is funded by Guangdong Science & Technology Infrastructure Construction Project (Number: 2019B030316004).

## Conflict of Interest

The authors declare that the research was conducted in the absence of any commercial or financial relationships that could be construed as a potential conflict of interest.

## Publisher’s Note

All claims expressed in this article are solely those of the authors and do not necessarily represent those of their affiliated organizations, or those of the publisher, the editors and the reviewers. Any product that may be evaluated in this article, or claim that may be made by its manufacturer, is not guaranteed or endorsed by the publisher.
